# Public awareness of corneal donation in an urban South African population

**DOI:** 10.4102/jcmsa.v4i1.443

**Published:** 2026-10-29

**Authors:** Faheema Hajee, Roland Hollhumer

**Affiliations:** 1 Department of Neurosciences, Division of Ophthalmology, University of the Witwatersrand, Johannesburg, South Africa

**Keywords:** organ donation, cornea, South Africa, corneal transplant, donor

## Abstract

**Background:**

Corneal pathology is the second leading cause of preventable blindness in sub-Saharan Africa. In most cases, a corneal transplant can improve vision. South Africa has an immense shortage of donor corneas. This study aimed to determine the public attitude towards corneal tissue donation in an urban South African population.

**Methods:**

This is an observational, cross-sectional quantitative study of the attitude of an urban South African population towards corneal tissue donation. A self-designed questionnaire was developed. The participants were asked about reasons for both willingness and unwillingness to donate their corneas.

**Results:**

A total of 100 participants (60 females and 40 males) completed the questionnaire. About 76% of participants were unaware of corneal donation. About 64% of participants were willing to donate their corneas, whereas 25% were not willing to do so. Cultural reasons were the primary reason respondents cited for being unwilling to donate their corneas. In this study, there was no statistically significant correlation between the willingness to donate corneal tissue and participants’ age, gender, level of education, and employment status. There was a statistically significant difference between the willingness to donate one’s solid organs and the willingness to donate one’s cornea (*p*-value 0.029). Participants were more willing to donate their solid organs than they were willing to donate their corneas.

**Conclusion:**

There is a decreased level of awareness of corneal tissue donation in this urban South African population.

**Contribution:**

Understanding the level of awareness of corneal transplantation, which can guide awareness campaigns.

## Introduction

### Background

Corneal pathology is a leading cause of preventable blindness in sub-Saharan Africa.^1^ Corneal disease is the cause of visual loss in 11% of blind or severely visually impaired children and 4% of blind adults in South Africa.^1^ Studies have shown that low vision is associated with the loss of ability to work, reduces the quality of life, and can trigger depression.^1^

In most cases, a corneal transplant or keratoplasty can restore or significantly improve vision. Reversing corneal blindness with a corneal graft can prove to be of great benefit to the patient, the family, and society.^2^ Studies have shown that corneal transplants not only restore vision, but also reduce pain caused by an injured or diseased cornea.^2^

Keratoplasty is a surgical technique in which the damaged or diseased cornea is replaced with donated corneal tissue. Advances in surgical techniques and surgeon expertise have resulted in improved visual outcomes with these procedures.^1^ Corneal transplantation is the most frequently performed transplant procedure worldwide.^3^

In recent years, the availability of locally sourced corneal tissue in South Africa has declined significantly. The shortage of corneal tissue supply is apparent in both the private and public sectors.^4^ For the majority of South Africans, imported corneas are not affordable. Patients in the public sector who require corneal transplants are often placed on long waiting lists, and it may be months or even years before a graft becomes available. Most recent published data from the Gauteng Cornea Eye Bank showed that there were 1884 persons in South Africa on a waiting list for a corneal graft in 2008, and an estimated 330 corneal transplant surgeries were performed that year.^5^ In contrast to the United States, which had no patients on their waiting list in 2008 and performed 41 652 corneal transplant surgeries during that year.^1^

There has been a progressive year-on-year decline in the number of corneal donors, from 565 per year in 2002 to 89 in 2016. As a result, there has been an 85.5% decrease in the number of corneal transplants per year using locally donated corneas. The reasons given for the progressive decline in donations were threefold: Legislative changes in South African mortuaries, rising rates of infectious diseases among donors, and a lack of public awareness.^1^ The rapid decline in corneal donors from 2005 to 2008 coincided with a legislative change affecting South African forensic mortuaries. Before 2006, the South African Police Services controlled and managed forensic mortuaries in the country. During this period, Eye Bank directors were privy to information about deceased persons in the mortuaries and could therefore identify potential donors and contact their next of kin.^1^ This made mortuaries the main source of corneal donors at that time. Currently, state forensic mortuaries are under the governance of the National Department of Health. This has brought additional confidentiality and ethical considerations that are governed by the *National Health Act* and *Protection of Personal Information Act*.^1^

South Africa currently adopts an ‘opt-in system’ for organ donation. Countries that adopt an ‘opt-out system’, which implies that all persons are presumed to be organ and tissue donors unless they explicitly state their wishes against it, have been shown to have significantly higher donation rates.^1^

A review of 55 published studies from 13 countries showed that the most commonly cited reason for not being a registered eye donor was a lack of awareness about eye donation and not knowing how to pledge one’s eyes for donation after death.^6^ Awareness about eye donation and knowledge about corneal transplantation is one of the modifiable factors associated with an increased willingness to donate.^6^

Currently in South Africa, the final decision to donate corneal tissue lies with the family members of the deceased, even if the deceased was a registered organ donor. International studies show family refusal rates for corneal tissue donation between 29% and 59%. The rate of corneal tissue donation refusal is higher than that of multiple organ donation.^7^ The most frequent reason for refusal is family members being unaware of the desire of the potential donor. Another frequently cited reason is a delay in the release of the body. Family members also have a lack of knowledge regarding the time required for donation, which leads them to believe that there will be a delay in the religious rituals.^7^ There is a specific unwillingness to donate eyes or the cornea. A previous study of an adult South African population showed respondents were more willing to donate solid organs than corneas.^8^

South Africa has an immense shortage of donor corneas, causing the burden of corneal disease requiring transplantation to rise continually. This study assesses the level of knowledge and current attitude towards corneal donation among an urban population of South Africans. In addition, factors that might influence the willingness and unwillingness to donate corneal tissue will be investigated.

## Research methods and design

A cross-sectional study using mixed methods was performed. Ethics approval was granted by the Human Research Ethics Committee of the University of the Witwatersrand (M200644).

A structured questionnaire was developed to gather information on attitudes towards corneal tissue donation and to identify factors that might affect corneal donation in an urban South African setting. The study was conducted at Saint John Eye Hospital in Soweto between November 2021 and December 2021.

The study participants were randomly invited from patients or escorts attending the outpatient clinic at Saint John Eye Hospital. Study information sheets were distributed, and all those who agreed to participate signed an informed consent form. South African-born adults aged ≥ 18 years were asked to complete a self-administered questionnaire. This covered knowledge of corneal donation and reasons for willingness and unwillingness to donate corneal tissue. Participants who spoke a language other than English and whose responses could not be translated by the nursing staff were excluded from the study.

The questionnaire covered sociodemographic details (age, gender, population group, ethnicity, religious denomination, level of education and occupation), donation awareness, willingness to accept or donate corneal tissue for transplantation (with reasons), and preference for an opt-in or opt-out system.

Participant confidentiality was ensured by omitting any identifiers from data sheets. Each participant was assigned a unique reference number to maintain anonymity. Data were stored on a password-protected computer.

Data were analysed using Statistical Package for the Social Sciences (SPSS) software (IBM Corp., released 201. IBM SPSS Statistics for Windows, Version 25.0, Armonk, NY, United States). Descriptive statistics, including frequencies and percentages, were calculated to summarise the categorical data. Parametric data were compared using an *independent t*-test and reported as mean ± standard deviation [s.d.] and non-parametric data were compared using the Wilcoxon signed-ranked test and reported as median ± Interquartile range (IQR). A *p*-value of < 0.05 was considered statistically significant.

### Ethical considerations

Ethical clearance to conduct this study was obtained from the University of the Witwatersrand Human Research Ethics Committee (No. R14/49).

## Results

A total of 100 participants (40.0% males and 60.0% females) completed the questionnaire. The mean age of the study participants (±s.d.) was 41.26 (±14.23) years, ranging from 20 years to 80 years. Demographics of all participants are shown in Table 1.

**TABLE 1:** Participants’ sociodemographic characteristics (*N* = 100).

Variable	Number of respondents (%)	
	*n*	%
**Gender**		
Male	40	40.0
Female	60	60.0
**Race**		
Black people	95	95.0
White people	3	3.0
Mixed race people	2	2.0
**Ethnicity**		
Zulu	31	31.0
Sotho	25	25.0
Setswana	21	21.0
Sipedi	2	2.0
Venda	2	2.0
Xhosa	8	8.0
Ndebele	1	1.0
Swati	4	4.0
Tsonga	2	2.0
Other	4	4.0
**Religion**		
Christianity	90	90.0
Traditional African	9	9.0
Jewish	1	1.0
**Highest level of education**		
Primary	6	6.0
Secondary	68	68.0
Tertiary	25	25.0
**Occupation**		
Unemployed	29	29.0
Employed	53	53.0
Student	4	4.0
Pensioner	14	14.0

About 76% of participants had not previously heard of corneal donation. After being informed about corneal donation, willingness to donate corneas after death was confirmed by 64.0% of participants, whilst 25.0% were unwilling and 11.0% were unsure. Characteristics of participants willing to donate their corneas and the influence of study variables on willingness to donate their corneas are shown in Table 2. Age, gender, level of education, and status of employment did not significantly influence participants’ willingness to donate their corneas after death (*p* > 0.05).

**TABLE 2:** Characteristics of participants willing to donate their corneas and the influence of study variables on willingness towards corneal donation.

Demographics	Willingness to donate cornea		
	Yes	No	Unsure
**Total**	64	25	11
**Age (years)**			
18–29	16	7	2
30–50	35	9	7
> 50	13	9	2
**Gender**			
Male	24	9	7
Female	40	16	4
**Education**			
Primary	4	3	0
Secondary	46	14	8
Tertiary	14	8	3
**Employment**			
Employed	36	12	5
Unemployed	24	7	2
Pensioner	6	8	0

The participants who were unwilling to be corneal tissue donors cited the following reasons for refusal: About 53% were unwilling due to cultural reasons, 33% cited fear of disfigurement, 10% cited religious reasons, and 3% cited fear of organ trade (Figure 1).

**FIGURE 1:**
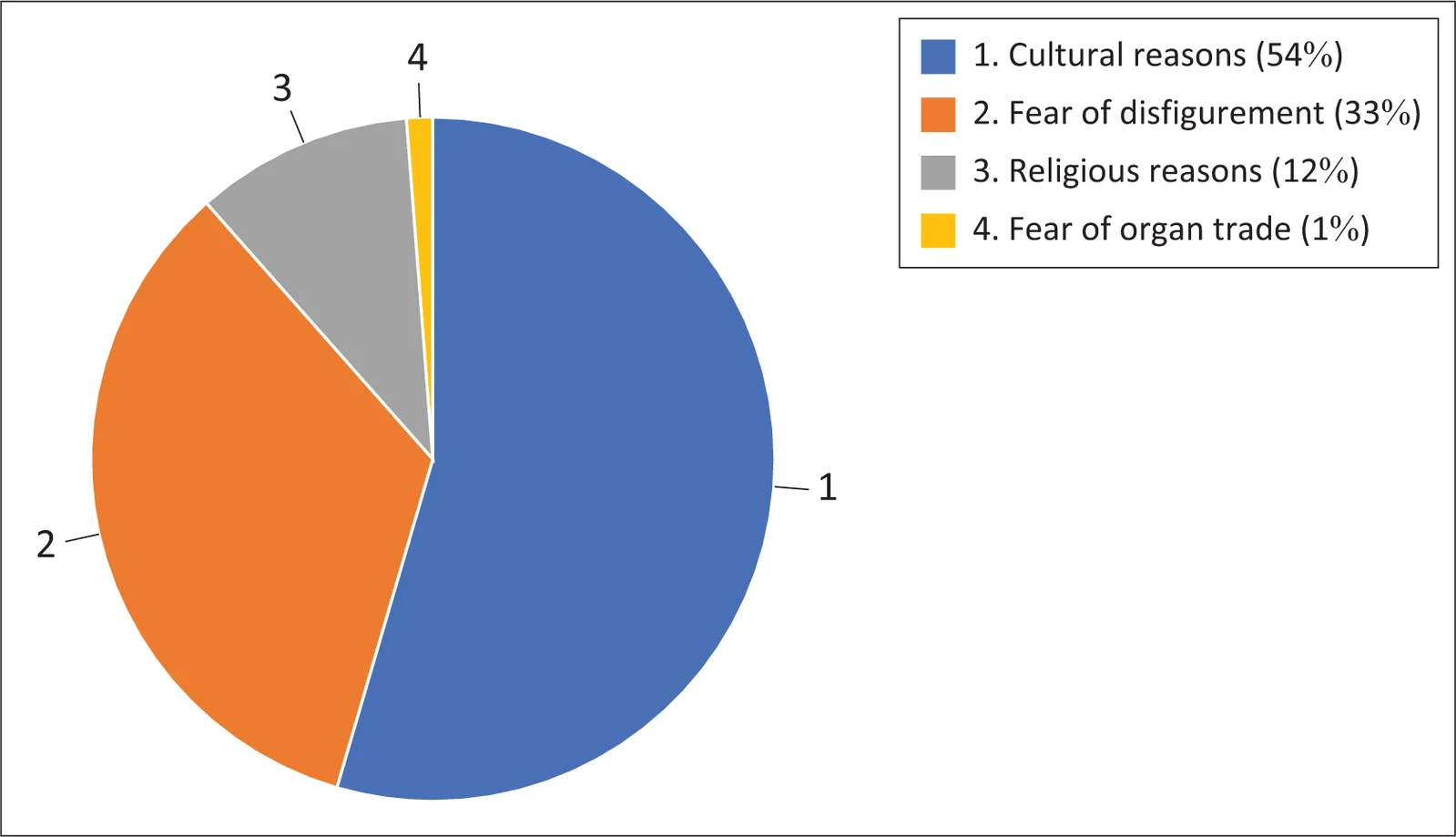
Reasons for unwillingness to donate the cornea after death.

There was a statistically significant difference between the willingness to donate one’s solid organs and the willingness to donate one’s cornea (*p* = 0.029). About 69% were willing to be organ donors, 19% were unwilling, and 12% were unsure.

The response to whether participants were willing to donate the cornea of their family members (who have expressed their will to be a corneal tissue donor prior to death) was as follows: 84% of participants were willing to go ahead with their family members’ request, 13% were unwilling, and 3% were unsure.

The response to whether participants were willing to donate the solid organs of their family members (who have expressed their will to be an organ donor prior to death) were as follows: About 84% were willing to fulfil the wishes of the deceased and agreed to have their family members solid organs donated, 12% were not agreeable to donating their family members organs, and 4% were unsure.

Responses about willingness to accept a corneal transplant were investigated by asking participants whether they would accept one if they required it to improve their vision. About 80% of participants responded that they would accept a corneal transplant. About 12% responded negatively, and 8% were unsure.

About 69% of participants favoured an opt-in system for organ and corneal tissue donation, while 31% favoured an opt-out system.

Participants’ responses regarding whether they perceived a shortage of corneal tissue donation in South Africa were as follows: 82% responded yes, there is a shortage of corneal tissue donation, 16% were unsure, and 2% responded no, there is no shortage of corneal tissue donation.

## Discussion

Corneal pathology is a leading cause of preventable blindness in sub-Saharan Africa. In most cases, a corneal transplant or keratoplasty can restore or significantly improve vision.^1^ In recent years, the availability of local corneal tissue in South Africa has been significantly reduced. The shortage of corneal tissue supply is apparent in both the private and public sectors. The reasons given for the progressive decline in donations were threefold: Legislative changes in South African mortuaries, rising rates of infectious diseases among donors, and lack of public awareness.^1^ For the majority of South Africans, imported corneas are not affordable. Patients in the public sector who require corneal transplants are often placed on long waiting lists, and it may be months or even years before a graft becomes available.

Several studies across different communities worldwide have shown that higher knowledge scores are associated with an increased willingness to donate. Similarly, lower levels of socio-economic status, formal education, and knowledge regarding corneal donation have been associated with a lower likelihood of believing the justification of corneal and organ donation and a lower likelihood of corneal donation.^6^ Our study showed a poor level of awareness and knowledge among the studied population. Most participants (76.0%) had not previously heard of corneal tissue donation. This is in keeping with other African studies where awareness of corneal transplantation was reported at 32.7% in a study conducted in Ghana and 9.9% in a study conducted in Nigeria.^9,10^ This contrasts with a study carried out in Jordan that reported a high level of public awareness of corneal donation at 88%.^11^ In our study, once participants were informed about corneal transplantation, more than half of the participants (64.0%) expressed willingness to donate their corneas after death. This highlights disparities in awareness profiles across countries and may indicate the effectiveness of ongoing public awareness campaigns.

In this study, there was no statistically significant correlation between the willingness to donate corneal tissue and participants’ age, gender, level of education and employment status. Several studies have shown that demographic factors such as the participants’ age, gender, and socio-economic status do not influence the willingness to donate the cornea.^12^ Other studies have shown only religion and ethnicity to have influenced the willingness to donate corneas.^13^ Although there were representations from all the African ethnic groups in this study population, most of the participants belonged to the Zulu (35.0%), Sotho (25.0%) and Setswana (21.0%) ethnic groups. About 90% of the participants were Christians. The study population was not representative of all the religions followed in South Africa. It was not possible in this current study to assess the correlation between the willingness to be a corneal tissue donor and ethnicity and religion.

Population groups with low donor rates may have cultural and other objections to tissue donation.^1^ Cultural factors regarding organ and tissue donation within other population groups and minority groups in South Africa have been extensively studied in previous studies.^1^ Within the Black African Culture, organ and tissue donation is a complex subject. The belief is that the deceased will join his or her ancestors in the afterlife and the body must remain whole for this transition. There is concern that if tissues or organs are removed, the ancestral spirits will be displeased and unable to rest. It is believed that the spirit may then return to those living to seek retribution for the removal of tissue and organs.^14^ Human organs have apparently been used by sangomas (traditional healers) to make muti (traditional medicine to ward off spells) and by ‘witches’, ‘wizards’ and ‘sorcerers’ for black magic. Although this is a separate issue, the association may adversely affect attitudes toward tissue donation.^14^ Our study showed that 53% of respondents cited cultural reasons for being unwilling to donate corneal tissue.

A study by Lawlor and Kerridge explored the reasons why individuals decide whether to donate corneas. The main concerns that contributed to refusal of corneal donation were disfigurement and loss of identity.^8^ About 33% of participants in our study said that they were fearful of disfigurement and would not donate for this reason.

Religion is an influential factor in the decision-making of organ and tissue donation. There are striking differences among the major faiths, but also a common theme: The dilemma between religious concerns and altruism.^7^ Our study showed this to be the reason for not being willing to donate in 10% of cases.

In this study, there was a statistically significant difference between the willingness to donate one’s solid organs and the willingness to donate one’s cornea. Participants were more willing to donate their solid organs than they were willing to donate their corneas. A previous study of an adult South African population showed respondents were more willing to donate solid organs than corneas.^8^ Approximately 30% of families in Australia who agree to multi-organ donation specifically refuse corneal donation, and a similar disparity is noted in large studies in the United States.^6^ The perceived physical alteration or disfigurement by removing the eyes or corneas of the deceased was a significant source of distress for family members. For some, there were concerns about the appearance of the body at a viewing; for others, the symbolism associated with the eyes raised concerns that removing them would diminish the human aspect of the deceased.^8^ In this study, most of the participants stated that they would fulfil the wishes of their deceased family members, with 84.0% of participants agreeing to their family members’ solid organ donation and 84.0% agreeing to their family members’ corneal tissue donation.

In the current study, 80.0% of participants were willing to accept a corneal transplant if needed. Of note, in this study, some participants were willing to accept a corneal transplant but were not willing to be corneal tissue donors. It has been widely reported that the demand for healthy corneas exceeds the actual supply of donated corneas.

South Africa currently adopts an ‘opt-in system’ for organ donation. Countries that adopt an ‘opt-out system’, which implies that all persons are presumed to be organ and tissue donors unless they explicitly state their wishes against it, have been shown to have significantly higher donation rates. Wales is the first country in the United Kingdom that has shifted to an opt-out system and has shown a 24% increase in donations in its first year.^1^ In this study, 69% of participants favoured an opt-in system for organ and corneal tissue donation, and 31% were in favour of an opt-out system. Implementing an opt-out system may not be straightforward in South Africa due to diverse cultural and religious beliefs. Another contributing factor is the level of awareness and education regarding organ and tissue donation. A change in this legislation, however, would prove beneficial not only for corneal tissue donation but also for other organ donations.^1^

## Conclusion

A limitation of this study is that the questionnaire was designed as a multiple-choice survey, with no opportunity for participants to provide open-ended answers. This limited the ability of this study to elaborate on specific reasons for unwillingness to donate corneal tissue. This study was based on an eye hospital-based sample of participants, which may limit extrapolation to the general population. The study population was not representative of all the religions followed in South Africa. It was not possible in this current study to assess the correlation between the willingness to be a corneal tissue donor and ethnicity and religion.

This study showed that participants demonstrated a decreased level of awareness about corneal tissue donation in South Africa. However, most participants were willing to donate their corneas after receiving information. The main reason for unwillingness to donate their corneas was cultural beliefs. This study highlighted that there is a specific unwillingness to donate eyes or corneas compared with the donation of solid organs. South Africa has an immense shortage of donor corneas, causing the burden of corneal disease requiring transplantation to rise continually. This study emphasised the need to raise awareness of corneal donation, which, in turn, will reduce misconceptions and increase the supply of local donor tissues.
